# Clarity and consistency in stillbirth reporting in Europe: why is it so hard to get this right?

**DOI:** 10.1093/eurpub/ckac001

**Published:** 2022-02-14

**Authors:** Mika Gissler, Mélanie Durox, Lucy Smith, Béatrice Blondel, Lisa Broeders, Ashna Hindori-Mohangoo, Karen Kearns, Rumyana Kolarova, Marzia Loghi, Urelija Rodin, Katarzyna Szamotulska, Petr Velebil, Guy Weber, Oscar Zurriaga, Jennifer Zeitlin, the Euro-Peristat Research Network, Gerald Haidinger, Jeannette Klimont, Sophie Alexander, Gisèle Vandervelpen, Wei-Hong Zhang, Evelin Yordanova, Rumyana Kolarova, Boris Filipovic-Grcic, Zeljka Drausnik, Urelija Rodin, Theopisti Kyprianou, Vasos Scoutellas, Petr Velebil, Laust Mortensen, Luule Sakkeus, Mika Gissler, Anna Heino, Béatrice Blondel, Anne Chantry, Catherine Deneux Tharaux, Guenther Heller, Nicholas Lack, Aris Antsaklis, István Berbik, Helga Sól Ólafsdóttir, Karen Kearns, Izabela Sikora, Marina Cuttini, Marzia Loghi, Cristina Tamburini, Serena Donati, Janis Misins, Irisa Zile, Jelena Isakova, Audrey Billy, Sophie Couffignal, Aline Lecomte, Guy Weber, Miriam Gatt, Peter Achterberg, Lisa Broeders, Ashna Hindori-Mohangoo, Jan Nijhuis, Rupali Akerkar, Kari Klungsøyr, Ewa Mierzejewska, Katarzyna Szamotulska, Henrique Barros, Mihai Horga, Vlad Tica, Jan Cap, Natasa Tul, Ivan Verdenik, Francisco Bolumar, Mireia Jané, Adela Recio Alcaide, Maria José Vidal, Oscar Zurriaga, Karin Källén, Anastasia Nyman, Sylvan Berrut, Mélanie Riggenbach, Tonia A Rihs, Alison Macfarlane, Lucy Smith, Rachael Wood, Jennifer Zeitlin, Mélanie Durox, Marie Delnord, Alice Hocquette

**Affiliations:** 1 THL Finnish Institute for Health and Welfare, Helsinki, Finland and Karolinska Institute, Stockholm, Sweden; 2 Université de Paris, CRESS, Obstetrical Perinatal and Pediatric Epidemiology Research Team (EPOPé), INSERM, INRA, Paris, F-75004, France; 3 Department of Health Sciences, College of Life Sciences, University of Leicester, LE1 7RH, UK; 4 The Netherlands Perinatal Registry (Perined), Utrecht, The Netherlands; 5 Foundation for Perinatal Interventions and Research in Suriname (PeriSur), Paramaribo, Suriname; 6 Tulane University, School of Public Health and Tropical Medicine, New Orleans, USA; 7 National Finance Division, Healthcare Pricing Office, HSE, Dublin; 8 Ministry of Health of Bulgaria, Sofia, Bulgaria; 9 Directorate for Social Statistics and Welfare, Italian Statistical Institute (ISTAT), Rome, Italy; 10 Croatian Institute of Public Health, School of Public Health ‘Andrija Štampar’, School of Medicine, University of Zagreb, Zagreb, Croatia; 11 Department of Epidemiology and Biostatistics, National Research Institute of Mother and Child, Warsaw, Poland; 12 Institute for the Care of Mother and Child, Prague, Czech Republic; 13 Department of Epidemiology and Statistics, Directorate of Health, Luxembourg; 14 Public Health General Directorate, Valencia Regional Public Health Authority, Spain; 15 Public Health and Preventive Medicine Department, University of Valencia, Spain; 16 Centre for Network Biomedical Research in Epidemiology and Public Health (CIBERESP), Madrid, Spain

## Abstract

**Background:**

Stillbirth is a major public health problem, but measurement remains a challenge even in high-income countries. We compared routine stillbirth statistics in Europe reported by Eurostat with data from the Euro-Peristat research network.

**Methods:**

We used data on stillbirths in 2015 from both sources for 31 European countries. Stillbirth rates per 1000 total births were analyzed by gestational age (GA) and birthweight groups. Information on termination of pregnancy at ≥22 weeks’ GA was analyzed separately.

**Results:**

Routinely collected stillbirth rates were higher than those reported by the research network. For stillbirths with a birthweight ≥500 g, the difference between the mean rates of the countries for Eurostat and Euro-Peristat data was 22% [4.4/1000, versus 3.5/1000, mean difference 0.9 with 95% confidence interval (CI) 0.8–1.0]. When using a birthweight threshold of 1000 g, this difference was smaller, 12% (2.9/1000, versus 2.5/1000, mean difference 0.4 with 95% CI 0.3–0.5), but substantial differences remained for individual countries. In Euro-Peristat, missing data on birthweight ranged from 0% to 29% (average 5.0%) and were higher than missing data for GA (0–23%, average 1.8%).

**Conclusions:**

Routine stillbirth data for European countries in international databases are not comparable and should not be used for benchmarking or surveillance without careful verification with other sources. Recommendations for improvement include using a cut-off based on GA, excluding late terminations of pregnancy and linking multiple sources to improve the quality of national databases.

## Introduction

Recent work by international collaborations has called attention to the health burden associated with stillbirth and to the absence of, or very slight declines, in stillbirth rates in high-income countries.^1–4^ These reports have also revealed high heterogeneity in stillbirth rates across countries with comparable standards of living and health systems. Rates of stillbirth are over twice as high in those countries with the highest rates compared with those with the lowest.^3^ Since the causes of up to one-half of stillbirths remain unknown and many are associated with sub-optimal health care,^5^^,^^6^ it is essential to improve the availability and quality of data on these deaths to tackle this important health problem and identify the levers for achieving continuous improvement.

Despite longstanding registration of births and deaths in vital statistics and birth registries and recommendations by the World Health Organization (WHO) to collect data on stillbirths from 22 weeks’ gestational age (GA), many countries lack complete and reliable data on stillbirths, especially early stillbirths.^7–9^ Globally, differences between countries in criteria and practices for recording stillbirths render international benchmarks unreliable for fetal deaths before the third trimester of pregnancy. This limitation is acknowledged by the WHO which has recommended international comparisons of stillbirth rates only for births with a birthweight of ≥1000 g or, in more recent work, ≥28birthweeks’ GA.^4^

The comparability of stillbirth rates in Europe has been investigated by the Euro-Peristat network, a European research network that aims to improve monitoring and reporting of perinatal health indicators and that periodically compiles data using a common protocol in 31 European countries. Data on births and fetal deaths are collected by GA and birthweight groups which allow the application of denominators based on both these criteria. This also makes it possible to harmonize the population studied, despite the differences between countries in Europe in thresholds for recording stillbirth.^10^^,^^11^ Euro-Peristat has also collected information about terminations of pregnancy (TOPs), which are inconsistently reported and can have a strong impact on rates and trends, especially at early gestations.^12^^,^^13^

Analyses of these data have quantified the extent to which these differences influence stillbirth rates and thus their comparability across countries.^9^ Euro-Peristat also illustrated the importance of improving data on early stillbirths by showing that up to 27% of all stillbirths with a birthweight of 500 g or more occur between 500 and 999 g and 33% occur between 22 and 27 weeks’ GA.^10^ So, while the exclusion of early stillbirths may improve comparability, it substantially underestimates the health burden and creates discrepancies with neonatal mortality which is measured for live births of any gestation. In recent analyses, Euro-Peristat concluded that stillbirth rates could be reported for births at 24 weeks’ GA and over with good reliability^8^ although this could not currently be extended to stillbirths at 22 and 23 weeks’ GA.^14^

Euro-Peristat only collects new data periodically,^3^ and therefore data to assess annual trends in stillbirth in Europe must be taken from Eurostat, the official statistical system for demographic and health data in Europe based on data reported by national statistics offices. In 2011, Eurostat published implementing regulations for stillbirth reporting as part of regulation governing reporting of causes of death (EU 328/2011)^15^ under the regulation on community statistics on public health and health and safety at work (EU 1338/2008 with amendment EU 2019/1700).^16^ This regulation specifies that stillbirths be reported principally by birthweight criteria and that data be provided separately for stillbirths weighing 500–999 g and ≥1000 g. Providing data on stillbirths by year is compulsory, but data by birthweight group is voluntary. A separate department at Eurostat collects demographic statistics and includes information on stillbirths ≥28 weeks.

Given the importance of good quality data on stillbirths for health monitoring, we sought to assess the concordance between Eurostat routine statistics and data collected within the Euro-Peristat research network where quality checks based on GA and birthweight limits are applied.

## Methods

Data on stillbirths and live births were abstracted from Eurostat databases and compared with birth data collected for the Euro-Peristat project for the year 2015.

Thirty-one countries participated in the Euro-Peristat data collection (28 EU Member States at the time, Iceland, Norway and Switzerland). In most countries, data on stillbirths were taken from medical birth registers or demographic statistics, with largely mandatory provision and good coverage.^10^^,^^17^ When there were several birth data sources, the country team decided which was the most reliable. Euro-Peristat collects data on births ≥22 weeks’ GA or when GA is missing, with a birthweight ≥500 g. When countries cannot provide data using this definition, they use their national definition and specify this (see Supplementary Appendix for sources and criteria). Data on live births and stillbirths are collected for each gestational week and also by birthweight in 500-g intervals with options for specifying the number of births with missing data on GA or birthweight. Data are collected for stillbirths and TOPs separately when countries are able to distinguish between these outcomes.

As part of its Causes of Death Statistics, Eurostat compiles annual data on stillbirths for the 31 countries included in Euro-Peristat.^18^ Data are based on each country’s national definition. As specified in regulations,^15^ stillbirths are also collected in two groups: (i) stillbirths with birthweight from 500 to 999 g or (when birthweight does not apply) GA from 22 to 27 weeks, or (when neither of the two applies) crown heel length from 25 to 34 cm and (ii) stillbirths with birthweight ≥1000 g or (when birthweight does not apply) GA ≥28 completed weeks, or (when neither of the two applies) crown heel length ≥35 cm. National definitions for stillbirth were abstracted from meta-data files^19^ to create Supplementary table S1. Verification of the accuracy of these definitions was not undertaken.

Stillbirth rates are also reported in Eurostat’s European Demographic Statistics defined as late fetal deaths at 28 weeks or over.^20^ However, unlike the Cause of Death statistics, there is no legislated data collection guidelines for stillbirth and the instructions provided in the most recent 2015 Demographic Statistics manual do not specify GA reporting criteria.^21^ Therefore, although we included these data, we conducted our primary analysis on Cause of Death data. Data on live births were taken from demographic statistics, as live births are not collected for Cause of Death statistics.

Stillbirth rates were calculated per 1000 live and stillbirths. Since we did not have data on live births by birthweight or GA for Eurostat, we used all live births for the calculation of total births for both data sources regardless of the cut-off used for stillbirths. As very early live births <500 g, <1000 g or <28 weeks are infrequent (<1% of births), this imprecision in the denominator will have a minor effect on stillbirth rate estimates, although variation may be higher in smaller countries. We separately calculated stillbirth rates including and excluding late TOPs (≥22 weeks) when data were available. Using Euro-Peristat data, we also calculated the proportion of stillbirths with missing data on birthweight and on GA. For each country, we computed absolute and relative differences in stillbirth rates from both sources for all reported stillbirths and using 500-g, 1000-g and 28-week thresholds. For these analyses, Euro-Peristat was considered as the reference.

## Results

Table 1 provides data on live births and stillbirths for the two data sources. The number of total births ranged from a little over 4000 in Iceland and Malta to over 700 000 in Germany, France and the UK. Estimates were similar between Eurostat and Euro-Peristat, with most discrepancies totaling less than 1%. One exception was Luxembourg (11%). Stillbirths are presented as all reported stillbirths and using thresholds of 500 and 1000 g. For Euro-Peristat, most countries used the Euro-Peristat definition of ≥22 weeks for all stillbirths, unless this was not possible, as detailed in the Supplementary Appendix. For Eurostat, definitions, abstracted from meta-data files, are presented in Supplementary table S2. Some countries were missing Eurostat stillbirth data for birthweight groups in 2015, even though data were available for adjacent years (for instance, Italy had data for 2016 and 2017, Sweden for 2017 and Iceland for 2013 and 2014). Several countries provided data only by GA to Euro-Peristat (Denmark and Portugal) so we used 22 weeks as a proxy for 500 g and 28 weeks for 1000 g.

**Table 1:** Number of live births and fetal deaths and TOPs, Eurostat Cause of Death Statistics and Euro-Peristat data collection, 2015 unless noted

Data source	Eurostat				Euro-Peristat							
	Live births	**Stillbirths**			Live births	**Stillbirths**				**Terminations^d^**		
Country	Total^a^	Total^b^	≥500 g	≥1000 g	Total^c^	Total^c^	≥500 g	≥1000 g	BW missing	Total	≥500 g	≥1000 g
Belgium	122 274	561	546	364	122 240	598	532	359	4	NA	NA	NA
Bulgaria (2014)	67 585	503	503	373	67 585	498	490	367	0	NA	NA	NA
Czech Republic	110 764	540	539	322	110 764	398	392	274	4			
Denmark	58 205	208	195	116	57 677	170	–	–	–	24	NA	NA
Germany	737 575	2787			725 937	2559	2435	1630	0	NA	NA	NA
Estonia	13 907	52	52	38	13 907	54	50	39	0	0	0	0
Ireland	65 536	193	193	139	65 623	290	258	189	0	0	0	0
Greece	91 847	311	309	249	91 847	312	299	243	3	–	–	–
Spain	418 432	1309	1250	1016	420 283	1309	1098	897	204	0	0	0
France^e^	759 099^f^	6633	6633	3347	759 099	3824	2416	1616	855	2824	1924	885
Croatia	37 503	176	176	114	37 252	176	160	112	0	–	–	–
Italy	485 780	1390			484 777	1780	1289	1067	458	485	NA	NA
Cyprus	9170	30	25	13	9394	31	18	10	9	NA	NA	NA
Latvia	21 979	170	170	113	21 720	106	102	70	0	–	–	–
Lithuania	31 475	126	123	85	31 475	126	115	84	3	0	0	0
Luxembourg	6115	50	50	26	6832	30	24	14	1	27	24	12
Hungary	92 135	474	474	314	91 680	526	392	311	118	47	NA	NA
Malta	4325	18	18	9	4435	18	14	8	0	0	0	0
Netherlands	170 510	512		384	168 425	809	575	345	49	NA	NA	NA
Austria	84 381	290	290	194	83 607	277	277	188	0			
Poland (2014)	375 160	1345	1345	887	375 647	1 321	1321	887	0	0	0	0
Portugal	85 500	299	263	202	85 762	286	–	–	–	–	–	–
Romania	201 995	736	736	687	201 023	737	735	688	0	–	–	–
Slovenia	20 641	124	124	67	20 273	63	59	41	0	61	60	26
Slovakia	55 602	183	183	176	55 615	209	200	192	8	0	0	0
Finland	55 472	171	171	118	55 588	171	145	105	6	73	NA	NA
Sweden (2014)	114 907	464			115 246	464	389	323	11	0	0	0
UK^g^	776 746	3432	3336	2270	771 652	3545	2849	2085	76	884	602	204
Iceland	4 129	10			4088	10	8	8	0	0	0	0
Norway	58 815	237	237	136	59 711	218	170	130	6	3	NA	NA
Switzerland (2014)	85 287	379	379	196	84 891	315	255	169	2	57	38	7

a Live births from Eurostat demographic statistics; stillbirths from Cause of Death statistics.

b Country stillbirth definitions for Eurostat are in Supplementary table S1.

c Euro-Peristat collects data at births ≥22 weeks’ GA, but if not possible, local definitions are accepted, see Supplementary Appendix.

d Where blank, there are no TOP or very small number because: TOPs are not performed at all or not after 21 weeks, they are not registered at all or they are registered in another database. Where NA, TOPs are included as stillbirths, but information on the number of TOP is not available.

e Metropolitan France.

f Data taken from Euro-Peristat because data not available on Metropolitan France.

g UK data on live births from Euro-Peristat is the sum of live births from England and Wales, Scotland and Northern Ireland (see Supplementary Appendix).

The extent of differences between the two sources in the number of stillbirths varied widely between countries. The number of stillbirths was exactly or almost (±5 cases) the same for 12 countries. In contrast, Ireland reported over 50% more cases to Euro-Peristat compared with Eurostat, while the reverse was true for Luxembourg, France and Slovenia which reported >40% cases to Eurostat compared with Euro-Peristat. Information on birthweight was missing for 8.7% of all stillbirths (1817/20 774). The country average was 5.0% and the highest proportions of missing values were observed for Cyprus (29%), Italy (26%), Hungary and France (both 22%) and Spain (16%). Twelve countries reported no missing cases.

Table 1 also presents Euro-Peristat data on the number of TOPs. Several countries cannot distinguish terminations in overall stillbirth data (Belgium, Cyprus and the Netherlands), whereas other countries do not record terminations (Germany). Some countries do not have terminations because they are not authorized or are rare at or after 22 weeks (Croatia, Estonia, Iceland, Ireland, Lithuania, Malta, Poland, Slovakia, Spain and Sweden).

**Figure 1:**
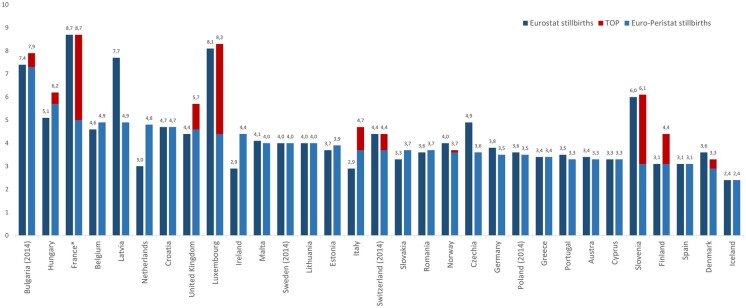
Stillbirth rate per 1000 total births in Eurostat cause of death statistics and Euro-Peristat by country in 2015, distinguishing between stillbirths and TOP and sorted by rates of stillbirth using Euro-Peristat data (solid red bar).

Figure 1 illustrates substantial differences in overall stillbirth rates from the two sources. Countries are sorted based on the Euro-Peristat stillbirth rate, without including terminations, although how the addition of terminations affects rates is indicated by a separate bar. This illustrates that the inclusion of terminations in Eurostat stillbirth statistics is one reason for the observed differences in rates, particularly affecting France, Luxembourg and Slovenia. Of note, Finland reports terminations to Euro-Peristat, but does not include them in stillbirth statistics provided to Eurostat. Other differences which are not due to terminations can also be observed, for instance in Hungary, Ireland and Latvia.

Tables 2 and 3 provide stillbirth rates using a lower limit of 500 and 1000 g, respectively. For stillbirths with a birthweight of 500 g or more, the difference between the averages for Eurostat data (4.4/1000, Standard Deviation (SD) 1.7) and Euro-Peristat data (3.5/1000, SD 0.9) was pronounced, due in part to terminations, as seen previously. Differences in stillbirth rates for birthweight of 1000 g or more were smaller between Eurostat data (2.9/1000, SD 0.9) and Euro-Peristat data (2.5/1000, SD 0.8) (table 3), but substantial differences existed for individual countries.

**Table 2:** Stillbirth rate ≥500 g for 31 European countries, Eurostat and Peristat 2015

Country	Eurostat cause of death	Euro-Peristat	Rate difference (95% CI)	Percentage difference (95% CI)
Belgium	4.4	4.3	−0.1 (−0.6, 0.4)	−3 (−14, 9)
Bulgaria (2014)	7.4	7.2	−0.2 (−1.1, 0.7)	−3 (−15, 10)
Czech Republic	4.8	3.5	−1.3 (−1.9, −0.8)	−27 (−38, −16)
Denmark	3.3	2.9	−0.4 (−1.0, 0.2)	−12 (−31, 7)
Germany	Missing	3.3		
Estonia	3.7	3.6	−0.1 (−1.6, 1.3)	−3 (−42, 34)
Ireland	2.9	3.9	1.0 (0.3, 1.6)	34 (12, 55)
Greece	3.4	3.2	−0.2 (−0.6, 0.4)	−6 (−19, 12)
Spain	3.0	2.6	−0.4 (−0.6, −0.1)	−13 (−20, −5)
France^a^	8.7	3.2	−5.5 (−5.7, −5.2)	−63 (−66, −61)
Croatia	4.7	4.3	−0.4 (−1.4, 0.6)	−9 (−29, 12)
Italy	Missing	2.6		
Cyprus	2.7	1.9	−0.8 (−2.2, 0.6)	−30 (−81, 21)
Latvia	7.7	4.7	−3.0 (−4.5, −1.5)	−39 (−58, −20)
Lithuania	3.9	3.6	−0.3 (−1.2, 0.7)	−8 (−31, 18)
Luxembourg	8.1	3.5	−4.6 (−7.3, −2.0)	−57 (−89, −24)
Hungary	5.1	4.3	−0.8 (−1.5, −0.2)	−16 (−29, −5)
Malta	4.1	3.1	−1.0 (−3.5, 1.5)	−24 (−85, 37)
Netherlands	Missing	3.4		
Austria	3.4	3.3	−0.1 (−0.7, 0.4)	−3 (−20, 13)
Poland (2014)	3.6	3.5	−0.1 (−0.3, 0.2)	−3 (−9, 6)
Portugal (≥22 weeks)	3.1	3.3	0.2 (−0.3, −0.8)	8 (−9, 26)
Romania	3.6	3.6	0.0 (−0.4, 0.4)	0 (−10, 11)
Slovenia	6.0	2.9	−3.1 (−4.4, −1.8)	−52 (−73, −30)
Slovakia	3.3	3.6	0.3 (−0.4, 1.0)	9 (−12, 30)
Finland	3.1	2.6	−0.5 (−1.1, 0.2)	−16 (−36, 5)
Sweden (2014)	Missing	3.4		
UK	4.3	3.7	−0.6 (−0.8, −0.4)	−14 (−19, −9)
Iceland	Missing	2.0		
Norway	4.0	2.8	−1.2 (−1.8, −0.5)	−30 (−46, −13)
Switzerland (2014)	4.4	3.0	−1.4 (−2.0, −0.9)	−32 (−45, −19)
				
Mean	4.4	3.5	−0.9 (−0.8, −1.0)	−11 (−13; −9)
Median	4.0	3.4	−0.4	−0
Minimum	2.7	1.9	−5.5	−63
Maximum	8.7	7.2	1.0	35

Euro-Peristat data for Denmark and Portugal refers to 22 weeks gestation or more.

a Metropolitan France only.

**Table 3:** Stillbirth rate ≥1000 g for 31 European countries, Eurostat and Euro-Peristat 2015

Country	Stillbirths per 1000 total births				
	Eurostat cause of death	Euro-Peristat	Rate difference (95% CI)	Percentage difference (95% CI)	
Belgium	3.0	2.9	0.0 (−0.5, 0.4)	−1 (−16, 13)
Bulgaria (2014)	5.5	5.4	−0.1 (−0.9, 0.7)	−2 (−16, 13)
Czech Republic	2.9	2.5	−0.4 (−0.9, 0.0)	−15 (−30, 0)
Denmark	2.0	2.0	0.0 (−0.5, −0.5)	0 (−26, 26)
Germany	Missing	2.2		
Estonia	2.7	2.8	0.1 (−1.2, 1.3)	3 (−43, 48)
Ireland	2.1	2.9	0.8 (0.2, 1.3)	36 (10, 61)
Greece	2.7	2.6	−0.1 (−0.5, 0.4)	−2 (−20, 15)
Spain	2.4	2.1	−0.3 (−0.5, −0.1)	−12 (−20, −4)
France^a^	4.4	2.1	−2.3 (−2.4, −2.1)	−52 (−56, −47)
Croatia	3.0	3.0	0.0 (−0.8, 0.8)	−1 (−27, 25)
Italy	Missing	2.2		
Cyprus	1.4	1.1	−0.4 (−1.4, 0.7)	−25 (−96, 47)
Latvia	5.1	3.2	−1.9 (−3.1, −0.7)	−37 (−61, −14)
Lithuania	2.7	2.7	0.0 (−0.8, 0.8)	−1 (−31, 29)
Luxembourg	4.2	2.0	−2.2 (−4.1, −0.2)	−52 (−98, −6)
Hungary	3.4	3.4	0.0 (−0.5, 0.5)	−1 (−16, 15)
Malta	2.1	1.8	−0.3 (−2.1, 1.6)	−13 (−102, 75)
Netherlands	2.2	2.0	−0.2 (−0.5, 0.1)	−9 (−23, 5)
Austria	2.3	2.2	−0.1 (−0.5, 0.4)	−2 (−22, 18)
Poland (2014)	2.4	2.4	0.0 (−0.2, 0.2)	0 (−9, 9)
Portugal	2.4	2.5	0.2 (−0.3, 0.6)	8 (−12, 27)
Romania	3.4	3.4	0.0 (−0.3, 0.4)	1 (−10, 11)
Slovenia	3.2	2.0	−1.2 (−2.2, −0.2)	−38 (−68, −7)
Slovakia	3.2	3.4	0.3 (−0.4, 1.0)	9 (−12, 30)
Finland	2.1	1.9	−0.2 (−0.8, 0.3)	−11 (−36, 14)
Sweden (2014)	Missing	2.8		
UK	2.9	2.7	−0.2 (−0.4, −0.1)	−8 (−13, −2)
Iceland	Missing	2.0		
Norway	2.3	2.2	−0.1 (−0.7, 0.4)	−6 (−29, 18)
Switzerland (2014)	2.3	2.0	−0.3 (−0.7, 0.1)	−13 (−32, 6)
Mean	2.9	2.5	−0.4 (−0.5, −0.3)	−12 (−15, −10)
Median	2.7	2.4	−0.1	−4
Minimum	1.4	1.1	−2.3	−52
Maximum	5.5	5.4	0.8	36

Euro-Peristat data for Denmark and Portugal refers to 28 weeks gestation or more.

a Metropolitan France only.

When applying the inclusion criteria of 500 g, the stillbirth rate declined most for France (−63%), Luxembourg (−57%), Slovenia (−52%), Latvia (−39%) and Switzerland (−32%). For Norway, Cyprus, Czech Republic, Malta, Finland, Hungary, UK, Spain and Denmark, the decline varied between −30% and −12%. Ireland (+34%) provided substantially higher rates for Euro-Peristat than for Eurostat.

Eurostat demography statistics showed larger disparities when compared with Euro-Peristat (Supplementary table S3). While data are reported as stillbirths at ≥28 weeks, this threshold does not seem to be applied in many countries, leading to substantial overestimation of rates. Comparisons with Euro-Peristat revealed that some countries provided data on all stillbirths instead of late stillbirths (Estonia, Germany, Latvia, Lithuania, Sweden, Switzerland and UK), while elsewhere other definitions were used (Czech Republic, Greece, Hungary, Italy and Norway). This table also presents Euro-Peristat data on stillbirths with missing GA. GA was missing less often than birthweight, with only Cyprus and Spain reporting proportions >5%.

## Discussion

Our comparison of stillbirth rates in Europe based on data reported by national statistical offices to the official European statistical office, Eurostat, and by the Euro-Peristat research network revealed substantial discrepancies between the two sources. These discrepancies remained even after adopting a common inclusion limit of 1000 g and ranged from rates that were 52% lower to 36% higher in Euro-Peristat compared with Eurostat; this range was even wider, −63% to +51% when a lower limit of 500 g was used. Differences of this magnitude affect benchmarking because they disrupt the order of country rankings. As a research network, which collects data using a standardized protocol with data quality checks, Euro-Peristat statistics are more comparable. Therefore, these differences are a cause for concern as Eurostat data represent official European statistics and are the only source of stillbirth data compiled annually for Europe. Eurostat also provides data to other international organizations, such as the OECD, which does not report stillbirth data, but uses these data to compute perinatal mortality statistics (stillbirths and early neonatal deaths) and to WHO which reports stillbirth rates from 1000 g.^22^

There are multiple reasons for inconsistency between these two data sources. Data provided to Eurostat come predominantly from demographic statistics collected by national statistical agencies, which may not collect information on birthweight or GA, whereas Euro-Peristat collects data from sources which have these data, such as medical birth registers and perinatal databases. Registration rules and criteria can also differ between data sources. In Ireland, for instance, Eurostat uses data from the Central Statistics Office which only includes registered births and perinatal deaths. In Ireland, it is not a legal requirement for parents/guardians to register stillbirths. Euro-Peristat uses data from the National Perinatal Reporting System, validated using hospital data by the Healthcare Pricing Office, which can provide data using Euro-Peristat definitions. For the UK, we used national birth registrations of live births from England and Wales, Scotland and Northern Ireland combined with stillbirth data for the UK from national perinatal mortality surveillance by MBRRACE-UK as this includes stillbirths starting at 22 weeks, whereas civil registration data only records stillbirths starting at 24 weeks.

Some countries combine data from several sources to improve completeness for Euro-Peristat, such as in Italy where data come from the spontaneous abortion register and civil registration data. A similar situation contributes to inconsistency between sources in the Netherlands, where stillbirths are also only registered from 24 weeks in civil registers, but data provided to Euro-Peristat come from linked clinical registers. Likewise, TOPs may not be identifiable in demographic data and these were major contributors to the discrepancies between Euro-Peristat and Eurostat. In the Euro-Peristat project, the country’s Scientific Committee member selects the highest quality population-based data with national coverage for describing perinatal indicators and these are usually birth registers or other medical data sources.^17^ In contrast, national statistical offices are responsible for reporting to Eurostat, so they do not have a choice and are bound by the limitations of legislation governing national statistics. In addition, stillbirths are usually reported to Eurostat as part of a much larger process of reporting all deaths or a wide range of demographic data.

Discrepancies can also result from population inclusion and exclusion criteria. Demographic statistics most often exclude cases where the mother is not a citizen or permanent resident, while medical birth registers and perinatal database include births in the country without applying any restrictions to citizenship or residents. For instance, in Luxembourg, this was the explanation for large discrepancies between births included in Euro-Peristat (*de facto* births: any birth occurring in Luxembourg) and those in Eurostat (*de jure* births: those to residents of Luxembourg only, and wherever the place of occurrence).^23^

Third, countries may not consistently follow requested definitions when providing data to Eurostat or the discrepancies could be due to errors. Euro-Peristat collects data by 500 g groups and by GA so that cross-checks are possible, but Eurostat only collects data already grouped together as provided by the statistical offices, so verification is difficult. Furthermore, Eurostat accepts data by GA if countries do not report stillbirth data by birthweight, but this is not explicitly noted in most cases. For instance, in France, stillbirth data by birthweight are not routinely produced because these rely on linkage between maternal and birth hospitalizations in hospital discharge data and incomplete linkage leads to missing data, as seen in table 1. Malta also provides data to Eurostat by GA. A final difference relates to the denominator for rates; Euro-Peristat collects data only on births at 22 weeks and over. In contrast, Eurostat has no GA threshold, so reporting is defined by countries’ own thresholds.

The 2011 implementation regulation on causes of death (EU 328/2011), following the EU regulation on the Community statistics on public health and health and safety at work (1338/2008) was an important improvement to stillbirth reporting as previously no information on stillbirths by birthweight groups was collected. However, problems remain. First, the sum of the two subgroups (500–999 and ≥1000 g) is not necessarily equal to the total number of stillbirths because stillbirths with birthweight below 500 g or GA < 22 weeks or crown heel length <25 cm might be recorded in the total number of stillbirths. Second, the implementation regulation is mandatory for the total number of stillbirths, but the more detailed information is voluntarily. Finally, there is no agreement across European countries on whether and how TOPs ≥22 weeks should be reported. It is preferable to present stillbirth data without terminations because of differences in screening and late termination policies between countries^24^ and the high impact that they can have on stillbirth rates.^13^^,^^25^^,^^26^

A more general issue to consider in evaluating current Eurostat rules is the use of birthweight over GA. Euro-Peristat recommends using GA to establish cut-off thresholds for stillbirth reporting since country regulations governing registration of stillbirths principally use GA and not birthweight.^27^ Furthermore, as growth restriction is a major cause of stillbirth, using birthweight underestimates third-trimester stillbirths.^9^ WHO has also recently changed its recommendations to use of GA as opposed to birthweight.^4^

Our study also addressed the issue of missing birthweight and GA data. Higher proportions of missing data for birthweight than GA among stillbirths may reflect practices for weighing stillbirths or for recording this information when there is a stillbirth. While birthweight is more straightforward to measure than GA and is usually more complete in most countries, GA data are well recorded in Europe, likely because of high uptake of early antenatal care and dating ultrasounds. Higher proportions of missing birthweight are another reason to prefer GA for reporting of stillbirth indicators. Our results also suggest that internationally agreed upon methods for imputing missing data are needed when reporting thresholds are used, as simply excluding cases with missing data artificially lowers rates.

One way to improve data and to ensure compatibility between data collection in countries with multiple data sources is to encourage linkage, in particular between vital statistics and medical birth registers. Several European countries link these data on a routine basis or for research, showing its technical feasibility, but these practices are far from universal.^28^ Birth registers tend to have higher quality data on the clinical conditions affecting stillbirths enabling evaluation of perinatal policies and studies show that linking data between medical and vital statistics registries improves the quality of information.^29^ Use of medical data sources can also make it possible to identify terminations. Consolidating multiple sources of data would also avoid problems of inconsistency in international reporting related to use of one source over the other. Finally, a general recommendation is that any perinatal data including information on GA and birthweight should be cross-checked to identify discrepancies caused by coding or reporting error. Improving the quality of national birth data is the best way to ensure high-quality international statistics.

The strength of our study is the careful compilation of validated population-based data from a large number of countries. The main limitation is use of aggregated data which meant that we could not cross-check cases across the sources. We also did not have information on the characteristics of the stillbirths which limited our ability to describe the clinical or social characteristics of discrepant cases. Furthermore, due to small annual births in some countries, the number of stillbirths was small, making it difficult to measure rates with high precision.

## Conclusion

The stillbirth rate is a key indicator of population health and offers vital signals about the quality of maternity care and the health of mothers and newborns. International comparisons are a powerful tool to encourage political and societal debate and motivate countries to improve their perinatal health and healthcare systems. However, basing such inferences on comparisons that are not valid or robust can lead to inappropriate conclusions regarding healthcare provision with potentially significant financial and social implications. Our study suggests that the current Eurostat regulations and procedures for stillbirth reporting should be updated to ensure that the burden of stillbirth is accurately captured in European statistics and to enable robust, valid and effective comparisons. This should be done in tandem with more frequent collection of data, including micro-data, through research networks, such as Euro-Peristat, that make it possible to carry out comprehensive analyses of stillbirth and validate routine data. Improvement of national data on stillbirths, including amending legislation to be compatible with WHO and combining information sources to optimize reporting, is needed for the full success of these European initiatives and to guide effective policy to prevent stillbirth.

## Supplementary data

Supplementary data are available at EURPUB online.

## Funding

The Euro-Peristat project received funding from the European Commission through the Health Programme (grant numbers 20101301 and 664691). The funders had no role in study design, data collection and analysis, decision to publish or preparation of the manuscript.

*Conflict of interest:* None declared.

Key points

- Measuring stillbirth rates is problematic even in high-income countries.
- Routinely collected stillbirth rates were higher than those reported by the research network.
- Routine stillbirth data for European countries in international databases can only be used for benchmarking or surveillance after careful verification with other sources.

## Supplementary Material

ckac001_Supplementary_Data

## References

[1] Flenady V , Wojcieszek AM, Middleton P, et al Stillbirths: recall to action in high-income countries. Lancet 2016;387:691–702. 26794070 10.1016/S0140-6736(15)01020-X

[2] Zeitlin J , Mortensen L, Cuttini M, et al; Euro-Peristat Scientific Committee. Declines in stillbirth and neonatal mortality rates in Europe between 2004 and 2010: results from the Euro-Peristat project. J Epidemiol Community Health 2016;70:609–15. 26719590 10.1136/jech-2015-207013 PMC4893141

[3] Zeitlin J , Alexander S, Barros H, et al; Euro-Peristat Scientific Committee. Perinatal health monitoring through a European lens: eight lessons from the Euro-Peristat report on 2015 births. BJOG 2019;126:1518–22. 31260601 10.1111/1471-0528.15857

[4] United Nations Inter-agency Group for Child Mortality Estimation (UN IGME). A Neglected Tragedy: The Global Burden of Stillbirths. New York, NY: United Nations Children’s Fund, 2020.

[5] Norris T , Manktelow BN, Smith LK, Draper ES. Causes and temporal changes in nationally collected stillbirth audit data in high-resource settings. Semin Fetal Neonatal Med 2017;22:118–28. 28214157 10.1016/j.siny.2017.02.003

[6] Sauvegrain P , Carayol M, Piedvache A, et al; The REMIP Investigator Team. Understanding high rates of stillbirth and neonatal death in a disadvantaged, high-migrant district in France: a perinatal audit. Acta Obstet Gynecol Scand 2020;99:1163–73. 32155659 10.1111/aogs.13838

[7] Lawn JE , Blencowe H, Waiswa P, et al; Lancet Stillbirth Epidemiology Investigator Group. Stillbirths: rates, risk factors, and acceleration towards 2030. Lancet 2016;387:587–603. 26794078 10.1016/S0140-6736(15)00837-5

[8] Smith LK , Hindori-Mohangoo AD, Delnord M, et al Quantifying the burden of stillbirths before 28 weeks of completed gestational age in high-income countries: a population-based study of 19 European countries. Lancet 2018;392:1639–46. 30269877 10.1016/S0140-6736(18)31651-9

[9] Mohangoo AD , Blondel B, Gissler M, et al; The Euro-Peristat Scientific Committee. International comparisons of fetal and neonatal mortality rates in high-income countries: should exclusion thresholds be based on birth weight or gestational age? PLoS One 2013;8:e64869. 23700489 10.1371/journal.pone.0064869 PMC3658983

[10] Euro-Peristat Project. European Perinatal Health Report. *Core Indicators of the Health and Care of Pregnant Women and Babies in Europe in 2015*. Available at: http://www.europeristat.com/index.php/reports/european-perinatal-health-report-2015.html. (25 January 2022, date last accessed).

[11] Kelly K , Meaney S, Leitao S, O’Donoghue K. A review of stillbirth definitions: a rationale for change. Eur J Obstet Gynecol Reprod Biol 2020;256:235–45. 33248379 10.1016/j.ejogrb.2020.11.015

[12] Blondel B , Cuttini M, Hindori-Mohangoo AD, et al; Euro-Peristat Scientific Committee. How do late terminations of pregnancy affect comparisons of stillbirth rates in Europe? Analyses of aggregated routine data from the Euro-Peristat Project. BJOG 2018;125:226–34. 28557289 10.1111/1471-0528.14767

[13] Joseph KS , Kinniburgh B, Hutcheon JA, et al Determinants of increases in stillbirth rates from 2000 to 2010. CMAJ 2013;185:E345–351. 23569166 10.1503/cmaj.121372 PMC3652963

[14] Smith LK , Blondel B, Zeitlin J, et al; Euro-Peristat Scientific C. Producing valid statistics when legislation, culture and medical practices differ for births at or before the threshold of survival: report of a European workshop. BJOG 2019;127:314–8. 31580509 10.1111/1471-0528.15971 PMC7003918

[15] European Commission. Commission Regulation (EU) No 328/2011 of 5 April 2011 implementing Regulation (EC) No 1338/2008 of the European Parliament and of the Council on Community statistics on public health and health and safety at work, as regards statistics on causes of death Text with EEA relevance. *Official Journal of the European Union.* 6.4. Brussels, Belgium: European Union, 2011.

[16] European Commission. Regulation (EC) No 1338/2008 of the European Parliament and of the Council of 16 December 2008 on Community statistics on public health and health and safety at work (Text with EEA relevance). *Official Journal of the European Union*. Brussels, Belgium: European Union, 2008.

[17] Gissler M , Mohangoo AD, Blondel B, et al; Euro-Peristat Group. Perinatal health monitoring in Europe: results from the EURO-PERISTAT project. Inform Health Soc Care 2010;35:64–79. 20726736 10.3109/17538157.2010.492923

[18] Eurostat. *Peri-neonatal Mortality by Age of Mother, by Residence and Occurrence*. Available at: https://appsso.eurostat.ec.europa.eu/nui/show.do?dataset=hlth_cd_aperro&lang=en (25 January 2022, date last accessed).

[19] Eurostat. *Causes of Death (hlth_cdeath), Reference Metadata in Single Integrated Metadata Structure (SIMS)*. Available at: https://ec.europa.eu/eurostat/cache/metadata/en/hlth_cdeath_esms.htm (25 January 2022, date last accessed).

[20] Eurostat. *Late Foetal Deaths by Mother’s Age (online data code: DEMO_MFOET)*. Available at: https://ec.europa.eu/eurostat/databrowser/view/demo_mfoet/default/table?lang=en (25 January 2022, date last accessed).

[21] Eurostat. Demographic statistics: a review of definitions and methods of collection in 44 European countries. Eurostat *Manuals and Guidelines.* Luxembourg, Europe: Eurostat, 2015.

[22] World Health Organization. World Health Data Platform/GHO/Indicators. Stillbirth rate (per 1000 total births*).* Available at: https://www.who.int/data/gho/data/indicators/indicator-details/GHO/stillbirth-rate-(per-1000-total-births (January 2020, date last accessed).

[23] Ministère de la Santé - Luxembourg Institute of Health. Surveillance de la Santé Périnatale au Luxembourg*: Rapport Sur Les Naissances 2014–2015–2016 et Leur Evolution Depuis 2001.* Available at: https://sante.public.lu/fr/publications/s/surveillance-sante-perinatale-lux-2014-2015-2016/surveillance-sante-perinatale-lux-2014-2015-2016.pdf

[24] Garne E , Khoshnood B, Loane M, et al; The EUROCAT Working Group. Termination of pregnancy for fetal anomaly after 23 weeks of gestation: a European register-based study. BJOG 2010;117:660–6. 20374608 10.1111/j.1471-0528.2010.02531.x

[25] Monier I , Lelong N, Ancel PY, et al Indications leading to termination of pregnancy between 22(+0) and 31(+6) weeks of gestational age in France: a population-based cohort study. Eur J Obstet Gynecol Reprod Biol 2019;233:12–8. 30544027 10.1016/j.ejogrb.2018.11.021

[26] van der Pal-de Bruin KM , Graafmans W, Biermans MC, et al The influence of prenatal screening and termination of pregnancy on perinatal mortality rates. Prenat Diagn 2002;22:966–72. 12424757 10.1002/pd.442

[27] Mohangoo AD , Buitendijk SE, Szamotulska K, et al; Euro-Peristat Scientific Committee. Gestational age patterns of fetal and neonatal mortality in Europe: results from the Euro-Peristat project. PLoS One 2011;6:e24727. 22110575 10.1371/journal.pone.0024727 PMC3217927

[28] Delnord M , Szamotulska K, Hindori-Mohangoo AD, et al; Euro-Peristat Scientific Committee. Linking databases on perinatal health: a review of the literature and current practices in Europe. Eur J Public Health 2016;26:422–30. 26891058 10.1093/eurpub/ckv231 PMC4884328

[29] Lain SJ , Hadfield RM, Raynes-Greenow CH, et al Quality of data in perinatal population health databases: a systematic review. Med Care 2012;50:e7-20–20. 21617569 10.1097/MLR.0b013e31821d2b1d

