# Correction to: Clarity and consistency in stillbirth reporting in Europe: why is it so hard to get this right?

**DOI:** 10.1093/eurpub/ckac042

**Published:** 2022-04-29

This is a correction to: Mika Gissler, Mélanie Durox, Lucy Smith, Béatrice Blondel, Lisa Broeders, Ashna Hindori-Mohangoo, Karen Kearns, Rumyana Kolarova, Marzia Loghi, Urelija Rodin, Katarzyna Szamotulska, Petr Velebil, Guy Weber, Oscar Zurriaga, Jennifer Zeitlin, the Euro-Peristat Research Network, Clarity and consistency in stillbirth reporting in Europe: why is it so hard to get this right?, *European Journal of Public Health*, Volume 32, Issue 2, April 2022, Pages 200–206, https://doi.org/10.1093/eurpub/ckac001

In the originally published version of this manuscript, the country labels in Figure 1 were ordered incorrectly.

This error has been corrected.

