# Supplementary material for: Clarity and consistency in stillbirth reporting in Europe: why is it so hard to get this right?
Source: Eur J Public Health. 2022 Feb 14;32(2):200–6. doi: 10.1093/eurpub/ckac001 (PMC8975542; doi:10.1093/eurpub/ckac001)
Supplement: ckac001_Supplementary_Data [file ckac001_supplementary_data.doc]

**Supplementary material**

Supplementary Table 1: Euro-Peristat data sources and capacity to provide stillbirth data using Euro-Peristat’s inclusion criteria

| Country | Source name, Institution | Inclusion criteria if not ≥22 weeks | Exclusion of terminations |
| --- | --- | --- | --- |
| Belgium | Civil registration/birth certificates, Statistics Belgium (Statbel) | ≥500 grams | No |
| Bulgaria (2014) | Vital Statistic, National center for public health and analysis, National Statistics Institute | ≥800 grams and/or 26 weeks |  |
| Czech Republic | Czech Statistical Office (CZSO) | ≥500 grams |  |
| Denmark | Medical birth register, National patient register, The Centralized Civil Register, The Danish Health Data authority, under the Danish Ministry of Health |  |  |
| Germany | IQTIG, Federal Institute for the Quality of Medical Care | ≥500 grams |  |
| Estonia | Estonian Medical Birth Register, Linked Data from EMSR (Medical Birth) and SPR (Causes of Death)  Report of a health care institution on maternal deaths and child health, National Institute for Public Health |  |  |
| Ireland | National Perinatal Reporting System (NPRS), Healthcare Pricing Office (HPO) at the Health Service Executive (HSE) |  |  |
| Greece | Hellenic Statistical authority |  |  |
| Spain | Vital Statistics, Spanish National Statistics Institute (INE) | ≥180 days |  |
| France | PMSI (French hospital discharge data), ATIH : Technical agency of hospitalization information |  |  |
| Croatia | Croatian Medical Birth Database, Croatian Public Health Institute |  |  |
| Italy | Birth certificates, Ministry of Health  Survey on hospital discharges for spontaneous abortion, National Institute of Statistics of Italy (ISTAT)  Survey on induced abortion  Hospital Discharge, Regional authorities and Ministry of Health |  |  |
| Cyprus | Birth Register, The Health Monitoring Unit, Cyprus ministry of health |  | No |
| Latvia | The Medical Birth Register and Register of Cause of Death, The Centre for Disease Prevention and Control of Latvia |  |  |
| Lithuania | Medical Date of Births,  Database of the Demographic Statistics, Central Statistical Office (Statistics Lithuania)  Causes of Death register, Institute of Hygiene Health Information Centre (HI HIC) |  |  |
| Luxembourg | Perinatal Health Monitoring System, Luxembourg Institute of Health |  |  |
| Hungary | Hungarian Central Statistical Office | ≥24 weeks |  |
| Malta | National Obstetrics Information System, National Mortality Register, Directorate for Health Information and Research |  |  |
| Netherlands | Perined, The Netherlands Perinatal Registry |  | No |
| Austria | Birth and cause of death statistics for infant death, Statistics Austria | ≥500 grams |  |
| Poland (2014) | Vital statistics, Central Statistical Office | ≥500 grams |  |
| Portugal | National Statistics - Live births and fetal deaths, National Statistics Institute (INE) / Department of Demographic and Social Statistics / Demographic Statistics Unit (INE/ DES/DM) |  |  |
| Romania | NCSIPH fetal/ neonatal/infant deaths, National Institute for Statistics data for fetal deaths. National Center for Statistics and Informatics in Public Health for fetal deaths as a modality to validate the cause of death |  |  |
| Slovenia | National Perinatal Information System of Slovenia, Institute of Public Health | ≥500 grams |  |
| Slovakia | NCZI SOR SON, National Health Information Center |  |  |
| Finland | Medical Birth Register, National Institute for Health and Welfare THL |  |  |
| Sweden (2014) | Medical Birth Register, The National Board of Health and Welfare |  |  |
| United Kingdom | Stillbirths: MMBRACE UK, University of Oxford and University of Leicester  Live births: Data provided separately for: England and Wales (Civil registration of births and deaths, Office for National Statistics), Northern Ireland (NIMATS Northern Ireland Maternity System, Health and Social, Care Northern Ireland), Scotland (SMR02 - Scottish Morbidity Record 02 (Maternity inpatients and Day Cases), Information Services Division of NHS National Services Scotland) |  |  |
| Iceland | The Icelandic Birth Registration (IBR) |  |  |
| Norway | Medical Birth Register of Norway, The Norwegian Institute of Public Health |  |  |
| Switzerland (2014) | BEVNAT, statistics of natural population change (vital statistics), Swiss Federal Statistical Office |  |  |

**Supplementary material**

**Supplementary Table 2: Country stillbirth definitions, Eurostat Cause of Death Statistics** (<https://ec.europa.eu/eurostat/cache/metadata/en/hlth_cdeath_esms.htm>, accessed 15 January 2020)

| **Country** | **(a) National definitions used for stillbirths and (b) characteristics (gestational age, weight, crown-heel length) that are collected** |
| --- | --- |
| Belgium | a) Belgium: all the Federate entities: Fœtal death with at least 500 g, or if the weight is unknown, at least 22 weeks b) Belgium: all the Federate entities : Weight and gestational age |
| Bulgaria | a) A stillborn child is the one who has not shown signs of life and its weight is 600 g and more grams at the completion of pregnancy and/or the pregnancy has continued at least 22 weeks. b) : Weight, gestational age, length of the foetus |
| Czech Republic | a) General definition of the term of stillbirth is not contained in the national legislation. Existing legal regulation, the Act No. 372/2011 Sb., on health services defines the foetus after abortion that is a foetus, which after the complete expulsion or extraction from its mother shows none of the signs of life and at the same time its birth weight is lower than 500 g, and in case that the weight cannot be measured if the pregnancy lasted less than 22 weeks. From this definition the definition of stillbirth is derived (the foetus, which after the complete expulsion or extraction from its mother shows none of the signs of life and at the same time its birth weight is 500 g and over, and in case that the weight cannot be measured if the pregnancy lasted 22 weeks and over. The definition of stillbirth along with the definition of live birth and all cases of abortion is stated only in guidelines for filling in a death certificate. The national number of stillbirths is adjusted to being in compliance with the Commission Regulation definition. Spontaneous abortions that meet the definition of stillbirth are added. b) Gestational age, weight, crown-heel are collected. |
| Denmark | a) Stillbirth is defined as all births after gestation of 22 weeks (≥22+0), where the child shows no signs of life at birth b) These informations are not collected on the death certificate but on a specific certificate for stillbirth. Information as weight, gestational age, length, head circumference, malformation, the circumference of the child’s abdomen |
| Germany | a) Child showed no sign of life and its weight is at least 500g.  b) Age of mother. Parity. Until 2013 also weight and crown-heel. |
| Estonia | a) >500g   b) Yes to all |
| Greece | a) The same as in the regulation 328/2011 described  b) Gestational age, weight |
| Spain | a) Stillbirth is the death prior to the complete expulsion or extraction of a viable product of conception from its mother. The foetus is viable when the weight is at least 500gr or has a gestational age of at least 22 weeks. b) The characteristics collected for stillbirths are the weight and the gestational age. |
| France | a) Child died before birth b) No information on stillbirths |
| Croatia | a) A stillborn child (stillbirth) is considered every child that, after a complete expulsion or extraction from its mother, does not show any evidence of life, that is, who neither breathed nor showed any other evidence of life, providing that the pregnancy lasted for 22 weeks or longer and that the child weighted 500 grams or more. Data source for given data on stillbirths is Croatian National Institute of Public Health.  b) We collect all of those characteristics. |
| Italy | a) In Italy there is a definition applied in the survey on spontaneous abortion: stillbirths are foetal deaths with a gestational age of more than 180 days. Nevertheless, the official information source used to report stillbirths that is Birth Delivery Certificate (Decree of Ministry of Health 349/2001), collects data about stillbirths irrespective of the duration of pregnancy. This information source collects, in addition, all the characteristics used to classify and group stillbirths (gestational age, weight, crown-heel) according to the Commission Regulation (EU) No 328/2011.  b) Gestational age, weight, crown-heel |
| Cyprus | a) For the data entry of stillbirths: We use the definition of the “COMMISSION REGULATION (EU) No 328/2011 of 5 April 2011 implementing Regulation (EC) No 1338/2008 of the European Parliament and of the Council on Community statistics on public health and health and safety at work, as regards statistics on causes of death”, where “stillbirth means foetal death, namely death prior to the complete expulsion or extraction from its mother of a product of conception, irrespective of the duration of pregnancy. Death is indicated by the fact that after such separation from its mother the foetus does not breathe or show any other evidence of life, such as beating of the heart, pulsation of the umbilical cord, or definite movement of voluntary muscles; For stillbirths at least one of three reporting criteria shall be applied in the following order: (a) birth weight from 500 g to 999 g or when birth weight does not apply gestational age from 22 to 27 completed weeks, or when neither of the two applies crown-heel length from 25 to 34 cm”. The National Legislation  “Law 168 (I) / 2017 LAW AMENDING THE LAW OF POPULATION LAW OF 2002 TO (NUMBER 4) OF 2015”, based on the COMMISSION REGULATION (EU) No 328/2011, has adjusted the definition of stillbirth such as "Stillbirth" means embryo death which is born with a weight of at least five hundred (500) grams or a gestational age of at least twenty-two (22) completed weeks or a body length from the top of the head to the heel of at least twenty-five (25) centimeters.  b) Up to 2013 we collected the weight and the gestational age; from 2014 some stillbirths which had a death certificate had the information of the crown-heel. Stillbirths without a death certificate are collected through the medical birth registry which does not include the information for crown-heel. |
| Latvia | a) Stillbirth – is foetus born lifeless after 22 weeks of pregnancy (after 154 days, when weight of foetus is 500 g, usually). Death is confirmed with fact that foetus is not breathing after separation from mother and showing no evidence of life as heart activity, pulsation of umbilical cord or motion of muscles.  b) We collect gestational age, weight and crown-heel. |
| Lithuania | a) Stillbirth means foetal death, namely death prior to the complete expulsion or extraction from its mother of a product of conception, irrespective of the duration of pregnancy. Death is indicated by the fact that after such separation from its mother the foetus does not breathe or show any other evidence of life, such as beating of the heart, pulsation of the umbilical cord, or definite movement of voluntary muscles.  b) not specified |
| Luxembourg | a) Actual national recommendation of Health Ministry based on WHO definition: fetal death is death prior to the complete expulsion or extraction from its mother of a product of conception, when stillbirth is at least 500 gr birth weight or 22 weeks of gestational age; the death is indicated by the fact that after such separation the fetus does not breathe or show any other evidence of life, such as beating of the heart, pulsation of the umbilical cord, or definite movement of voluntary muscles. Actual legal framework (décret du 4 juillet 1806 du code Napoléon) : all stillbirths of 6 months gestational age has to be declared b) Gestational age and weight |
| Hungary | a) If after the separation from the mother's body the foetus did not show any sign of life and if from the conception longer than 24 complete weeks passed or if the age of the foetus cannot be stated, the length of the foetus is 30 cm or more or if the weight of the foetus is 500 g or more, while in case of twin birth if at least one of the foetus' born alive.   b) Social-demographic information about the female (the mother) and the following: 1. Place of death (outside/in an institute); 2. When did the foetus die? (before delivery, in the phase of dilatation, in the phase of expulsion); 3. Date of death; 4. Gestational age, weight, crown-heel length; 5. Single or multiple foetus? Which in the sequence? 6. Sex; 7. Congenital malformations (2); 8. The way of starting of the delivery (spontaneous, induced delivery, programmed delivery, elective cesarean, other); 9. Cause of death: maternal conditions (2), foetal conditions (2); 10. Status of the child-bearing female (recovered, needs a further therapy, died); 11. Number of hospitalization days; 12. Date of discharge. (We collect similar but less information about early and medium foetal deaths. Since the national definition differs from Eurostat, some early and medium foetal deaths are also sent to Eurostat database.) |
| Malta | a) Deaths in foetuses from 22 weeks gestation onwards &/or 500g at birth or over. b) Gestational age, weight |
| Netherlands | a) A stillbirth is a fetus who was born lifeless after a gestational age of at least 24 weeks. Within this dataset only the stillbirth second group (gestational age of at least 28 weeks) are included. b) Gestational age |
| Austria | a) WHO-definition (500 grams or more and no signs of life)  b) gestational age, weight, crown-heel length |
| Poland | a) Still birth is death prior to the complete expulsion or extraction from mother of a product of conception, on condition that weighted at least 500 grams (or 22 weeks of gestational age); the death is indicated by the fact that after separation the foetus shows any signs of life, such as beating of the heart, pulsation of the umbilical cord or definite movement of voluntary muscles.  b) Up to 2014 the source of data of stillbirths was "Notification of birth" (live birth and stillbirth) and was collected: For newborn: data and place of birth, sex, weight, crown-heel, multiplicity, gestational age, place of delivery, number of previous live births or stillbirths, date of previous birth, moment of death, citizenship; For mother and father: data of birth, place of resident, PIN number (PESEL), marital status, data of marriage, educational level and source of maintenance. For the years 2015, 2016 and 2017 the source of data of stillbirths is a hospital's report containing general information only: weight and place (voivodship) of occurrence. |
| Portugal | a) A product of fertilisation whose death occurs before delivery or complete extraction from the mother's body, regardless of the duration of the pregnancy. Death is indicated by the fact that, after separation, the foetus has not breathed or shown any signs of life such as heartbeat, umbilical pulse or actual contractions of any muscles subject to voluntary action.  b) Gestational age, weight. |
| Romania | a) Late foetal deaths represent a product of conception completely extracted or ejected from the mother's body after at least 28 weeks of pregnancy, which after this separation gives no sign of life.  b) gestational age, weight and many other data items on demographic and social characteristics of parents (see website for details) |
| Slovenia | a) https://podatki.nijz.si/docs/3c_Fetalne_smrti_Metodolo%C5%A1ka_pojasnila_NIJZ.pdf (in Slovenian)  b) Gestational age, weight, crown-heel |
| Slovakia | a) A stillborn child means a child who did not show any signs of life at any time after being born.  b) For stillbirths, data collection is limited to the following groups:  (a) birth weight from 500 g to 999 g or when birth weight does not apply gestational age from 22 to 27 completed weeks, or when neither of the two applies crown-heel length from 25 to 34 cm; and (b) birth weight of 1 000 g and more or when birth weight does not apply gestational age after 27 completed weeks or when neither of the two applies crown-heel length of 35 cm or more. |
| Finland | a) Stillbirths include a fetus or a newborn who shows no signs of life at the time of birth and the duration of pregnancy is at least 22 weeks or the weight at least 500 grams. Miscarriages that occurred at an earlier stage of the pregnancy are not regarded as stillbirths and are not included in cause of death statistics. b) Gestational age and weight |
| Sweden | a) Up to and including June 2008: Death after 28 weeks of gestation. From July 2008: death after 22 weeks of gestation. b) Stillbirths are not included in the Cause of Death Register, they are registered in the Medical Birth Register but without any cause of death. In the Medical Birth Register variables such as gestational age, gestational weight, crown-heel, Apgar score, plurality, mode of delivery, sex, hospital etc. are collected for all deliveries in Sweden. Also information regarding the mother such as maternal age, country of birth, BMI, tobacco use, parity and diagnoses. |
| United Kingdom | a) England and Wales A stillbirth is defined as 'a child which has issued forth from its mother after the twenty-fourth (24th) week of pregnancy and which did not at any time after being completely expelled from its mother breathe or show other signs of life'. Scotland A stillbirth is defined as 'a child which has issued forth from its mother after the twenty-fourth (24th) week of pregnancy and which did not at any time after being completely expelled from its mother breathe or show other signs of life'. Northern Ireland A stillbirth is defined as 'a child which has issued forth from its mother after the twenty-fourth (24th) week of pregnancy and which did not at any time after being completely expelled from its mother breathe or show other signs of life'.  b) England and Wales: Gestational age, weight, Scotland: Gestational age, weight, Northern Ireland: Gestational age, weight |
| Ireland | a) WHO definition. b) Gestational age, weight. |
| Norway | a) Gestational age:≥ 22 weeks, weight: ≥500 g (According to the Medical Birth Registry (MBRN)  b) Medical Birth Registry in Norway (MBRN) collects gestational age, births weight ,crown heel, gender, plurality, congenital malformations and deformations |
| Switzerland | a) Until 2004, all stillborns with size equal to at least 30 cm. Since 2005, the inclusion criteria is a minimal gestation period of 22 completed weeks or a minimal birthweight of 500g.  b) Gestational age, birthweight and crown-heel |

Supplementary Table 3: Number of stillbirths and stillbirth rate at ≥ 28 weeks of gestation for 31 European countries, comparison between Eurostat demographic statistics and Euro-Peristat data for 2015

|  | Eurostat | Euro |  | | Stillbirths per 1000 total births | | |  | |
| --- | --- | --- | --- | --- | --- | --- | --- | --- | --- |
|  | Demographic statistics  ≥28 weeks | -Peristat  Stillbirths ≥28 weeks | Missing GA | | Eurostat  Demographic statistics | Euro-Peristat | Rate  Difference | | Percent  Difference |
| Belgium | Missing | 368 | 6 |  | | 3.0 |  | |  |
| Bulgaria (2014) | 498 | 388 | 0 | 7.3 | | 5.7 | -1.6 | | -22 |
| Czech Republic | 296 | 296 | 7 | 2.7 | | 2.7 | 0.0 | | 0 |
| Denmark | Missing | 115 | 0 |  | | 2.0 |  | |  |
| Germany | 2787 | 1759 | 63 | 3.8 | | 2.4 | -1.3 | | -36 |
| Estonia | 43 | 43 | 0 | 3.1 | | 3.1 | 0.0 | | 0 |
| Ireland | Missing | 222 | 0 |  | | 3.4 |  | |  |
| Greece | 272 | 270 | 2 | 3.0 | | 2.9 | 0.0 | | -1 |
| Spain | 1287 | 968 | 153 | 3.1 | | 2.3 | -0.8 | | -25 |
| France* | Missing | 2286 | 0 |  | | 3.0 |  | |  |
| Croatia | 163 | 119 | 0 | 4.3 | | 3.2 | -1.1 | | -27 |
| Italy | Missing | 1175 | 6 |  | | 2.4 |  | |  |
| Cyprus | Missing | 15 | 5 |  | | 1.6 |  | |  |
| Latvia | 72 | 73 | 0 | 3.3 | | 3.3 | 0.1 | | 3 |
| Lithuania | 126 | 90 | 3 | 4.0 | | 2.8 | -1.1 | | -29 |
| Luxembourg | 50 | 16 | 1 | 8.1 | | 2.3 | -5.8 | | -71 |
| Hungary | 408 | 338 | 0 | 4.4 | | 3.7 | -0.7 | | -17 |
| Malta | Missing | 11 | 0 |  | | 2.5 |  | |  |
| Netherlands | 500 | 358 | 35 | 2.9 | | 2.1 | -0.8 | | -28 |
| Austria | Missing | 202 | 0 |  | | 2.4 |  | |  |
| Poland (2014) | 928 | 932 | 3 | 2.5 | | 2.5 | 0.0 | | 0 |
| Portugal | 197 | 218 | 0 | 2.3 | | 2.5 | 0.2 | | 10 |
| Romania | 735 | 724 | 2 | 3.6 | | 3.6 | 0.0 | | -1 |
| Slovenia | 55 | 48 | 0 | 2.6 | | 2.4 | -0.3 | | -11 |
| Slovakia | 184 | 197 | 8 | 3.3 | | 3.5 | 0.2 | | 7 |
| Finland | 115 | 114 | 1 | 2.1 | | 2.0 | 0.0 | | -1 |
| Sweden (2014) | 456 | 350 | 1 | 4.0 | | 3.0 | -0.9 | | -23 |
| United Kingdom | 3 434 | 2289 | 5 | 4.4 | | 3.0 | -1.4 | | -33 |
| Iceland | Missing | 8 | 0 |  | | 2.0 |  | |  |
| Norway | 174 | 135 | 3 | 2.9 | | 2.3 | -0.7 | | -24 |
| Switzerland (2014) | 368 | 200 | 0 | 4.3 | | 2.3 | -1.9 | | -45 |
|  |  |  |  |  | |  |  | |  |
| Mean | -- |  | -- | 3.7 | | 2.8 | -0.8 | | -22 |
| Minimum | -- |  | -- | 2.1 | | 1.6 | -5.8 | | -71 |
| Maximum | -- |  | -- | 8.1 | | 5.7 | 0.2 | | 10 |

* Metropolitan France only
